# Crystal structure of 1,3-bis­[(*E*)-benzyl­idene­amino]­propan-2-ol

**DOI:** 10.1107/S2056989017004741

**Published:** 2017-03-31

**Authors:** Augusto Rivera, Ingrid Miranda-Carvajal, Jaime Ríos-Motta, Michael Bolte

**Affiliations:** a Universidad Nacional de Colombia, Sede Bogotá, Facultad de Ciencias, Departamento de Química, Cra 30 No. 45-03, Bogotá, Código Postal 111321, Colombia; b Institut für Anorganische Chemie, J. W. Goethe-Universität Frankfurt, Max-von Laue-Str. 7, 60438 Frankfurt/Main, Germany

**Keywords:** crystal structure, hydrogen bonding, C—H⋯π inter­actions, Schiff bases

## Abstract

In the title compound, C_17_H_18_N_2_O, the central carbon atom with the OH substituent and one of the (*E*)-benzyl­idene­amino substituents are disordered over two sets of sites with occupancies of 0.851 (4) and 0.149 (4). The relative positions of the two disorder components is equivalent to a rotation of approximately 60° about the C—N single bond. In the crystal, the mol­ecules are held together by O—H⋯N hydrogen bonds, forming simple *C*(5) chains along the *b*-axis direction. In addition, pairs of the chains are further aggregated by weak C—H⋯π inter­actions.

## Abstract 2

The mol­ecular and crystal structure of the title Schiff base derivative is reported. The crystal packing depends on O—H⋯N hydrogen-bonds, augmented by C—H⋯π inter­actions.

## Chemical context   

During the last decades, inter­est in Schiff bases and their complexes has been constant due to their extensive use for industrial purposes and also for their broad range of biological activities (Al Zoubi *et al.* 2016 ▸; Sahu *et al.* 2012 ▸; Da Silva *et al.*, 2011 ▸; Przybylski *et al.* 2009 ▸). The common structural feature of these compounds is the presence of a azomethine group (–*R*—C=N–), which can act as a hydrogen-bond acceptor or a ligand. To gain more insight into the structural and spectroscopic properties of this potentially polydentate ligand, we report herein the mol­ecular structure of the title compound.
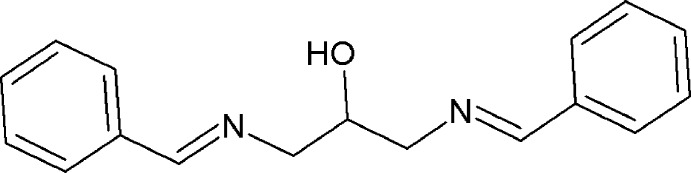


## Structural commentary   

**Figure 1:**
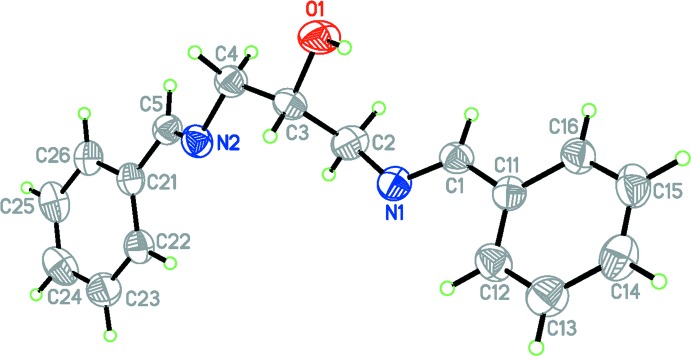
The mol­ecular structure of the title compound, Displacement ellipsoids are drawn at the 50% probability level. Only the major occupancy disorder component is shown.

The mol­ecular structure of the title compound is shown in Fig. 1 ▸. The compound exists in an *E,E* conformation with respect to the imine functions. One benzyl­idene­amino segment of the mol­ecule, C3/C4/N2/C5/C21–C26 is disordered over two sets of sites with a refined occupancy ratio of 0.851 (4):0.149 (4). The difference between the two conformers is reflected in the relative arrangement of the central spacer units. In the major disorder component, the torsion angle C3—C4—N2—C5 is −158.7 (2)° whereas the corresponding angle C3′—C4′—N2′—C5′ in the minor component is −93.3 (14)°. This translates to a rotation of approximately 60° about the C4—N2 bond. In the second, fully ordered, (*E*)-benzyl­idene­amino substituent, the equivalent torsion angles C1—N1—C2—C3 and C1—N1—C2—C3′ are −102.03 (18)° and −79.8 (8)°, respectively.

Unlike some related structures, which have a well-defined *synclinal (-sc)* alignment of the hydroxyl and imine nitro­gen atoms around the N(imine)—C—C—O(hydrox­yl) bond [−65.3 (3)° (Rivera, Miranda-Carvajal, Ríos-Motta & Bolte, 2016 ▸) and −67.6 (4)° (Moodley & Van Zyl, 2012 ▸)], the orientation between these groups in the title compound differs significantly, with the N1—C2—C3—O1 and N1—C2—C3′—O1 torsion angles being 81.51 (19)° and 21.2 (14)°, respectively.

The N1=C1 and N2=C5 distances in the mol­ecule are 1.270 (2) and 1.259 (3) Å, respectively, consistent with C=N double bonding. The C1—N1—C2 bond angle of 118.61 (15)° confirms the *sp* ^2^ character of N1. The bond angles C5—N2—C4 and C5′—N2′—C4′ [116.9 (2) and 114.6 (12)°, respectively] indicate a slight loss of the *sp* ^2^ character. The N1=C1 azomethine group is essentially co-planar with the attached benzene ring with an N1—C1—C11—C12 torsion angle being 2.0 (5)°. In contrast, in the disordered (*E*)-benzyl­idene­amino substituent, the corresponding torsion angles N2—C5—C21—C22 and N2′—C5′—C21′—C22′ are −17.6 (6) and 21 (4)° for the major and minor disorder components, respectively. All these data suggest that the difference between these (*E*)-benzyl­idene­amino substituents may result from some loss of conjugation between the phenyl ring and its azomethine substituent in the disordered branch of the mol­ecule.

## Supra­molecular features   

**Figure 2:**
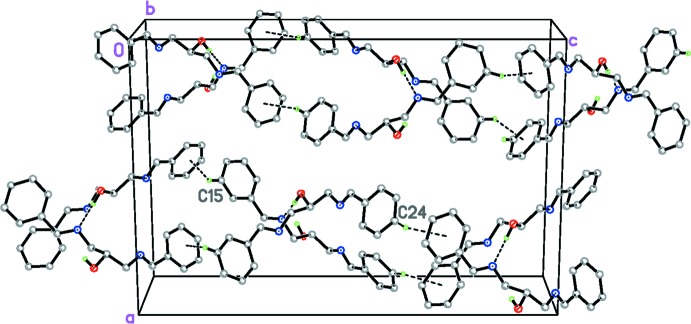
The crystal packing of the title compound showing the extended hydrogen-bonded network.

As found in related structures (Rivera, Miranda-Carvajal, Ríos-Motta & Bolte, 2016 ▸; Moodley & Van Zyl, 2012 ▸) in the crystal, mol­ecules are linked by an O1—H1⋯N1 hydrogen bond, Table 1 ▸, forming columnar structures built from *C*(5) chains along the *b*-axis direction. In addition, pairs of the chains are linked by weak C24—H24⋯*Cg*1 inter­actions (Table 1 ▸ and Fig. 2 ▸), involving the C11–C16 phenyl ring, together with C15—H15⋯*Cg*2 and C15—H15⋯*Cg*3 contacts involving the phenyl rings of the two disorder components; the centroids are defined in Table 1 ▸. It is noteworthy that the shortest (and presumably the strongest) of these non-classical contacts is C15—H15⋯*Cg*3 involving the phenyl ring in the minor disorder component (Table 1 ▸).

## Database survey   

A search in the Cambridge Crystallographic Database (CSD Version 5.38, last update 2016; Groom *et al.*, 2016 ▸) for the fragment 1,3-bis­[(benzyl­idene)amino]­propan-2-ol yielded the following structures: *N*,*N*′-[(2-hy­droxy-1,3-propanedi­yl)bis­(nitrilo­methylyl­idene-2,1-phenyl­ene)] bis­(4-methyl­benzene­sulfonamide) (Popov *et al.*, 2009 ▸), 2,2′-[(2-hy­droxy­propane-1,3-di­yl)bis(nitrilo­methylyl­idene)]diphenol (Azam, Hussain *et al.*, 2012 ▸), 1,3-bis­(2-hy­droxy-5-bromo­salicyl­idene­amine)­propan-2-ol (Elmali, 2000 ▸), 1,3-bis­[(*E*)-(2-chloro­benzyl­idene)amino]­propan-2-ol (Azam, Warad *et al.*, 2012 ▸) and 1,3-bis­[(*E*)-(4-meth­oxy­benzyl­idene)amino]­propan-2-ol (Rivera, Miranda-Carvajal, Ríos-Motta & Bolte, 2016 ▸). In each of these structures, the N=C double bonds adopt *E* conformations.

## Synthesis and crystallization   

The title compound was prepared as described by Rivera, Miranda-Carvajal & Ríos-Motta (2016 ▸). The crude product was recrystallized from diethyl ether solution with slow evaporation of the solvent, giving colorless crystals suitable for X–ray diffraction, m.p. 396.8–398 K, yield, 40%.

## Refinement   

Crystal data, data collection and structure refinement details are summarized in Table 2 ▸. The hydroxyl H atom was refined freely. All remaining H atoms were positioned geometrically and allowed to ride on their parent atoms, with *d*(C—H) = 0.95 Å for aromatic and azomethine atoms, *d*(C—H) = 0.99 Å for methyl­ene and *d*(C—H) = 1.00 Å for C3—H3. The *U*_iso_(H) values were constrained to 1.2*U*_eq_(C). The C3/C4/N2/C5/C21–C26 segment of the mol­ecule is disordered over two sets of sites with a refined occupancy ratio of 0.851 (4):0.149 (4).

## Supplementary Material

Crystal structure: contains datablock(s) I. DOI: 10.1107/S2056989017004741/sj5523sup1.cif

Structure factors: contains datablock(s) I. DOI: 10.1107/S2056989017004741/sj5523Isup2.hkl

Supporting information file. DOI: 10.1107/S2056989017004741/sj5523Isup3.cml

CCDC reference: 1540296

Additional supporting information:  crystallographic information; 3D view; checkCIF report

## Figures and Tables

**Table 1:** Hydrogen-bond geometry (Å, °) *Cg*1,*Cg*2, and *Cg*3, are the centroids of the C11–C16, C21–C26 and C21′–C26′ rings, respectively

*D*—H⋯*A*	*D*—H	H⋯*A*	*D*⋯*A*	*D*—H⋯*A*
O1—H1⋯N1^i^	0.93 (3)	1.92 (3)	2.8430 (19)	174 (2)
C24—H24⋯*Cg*1^ii^	0.95	2.88	3.802 (5)	164
C15—H15⋯*Cg*2^iii^	0.95	2.96	3.796 (3)	148
C15—H15⋯*Cg*3^iii^	0.95	2.79	3.640 (12)	150

Symmetry codes: (i) 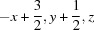; (ii) 
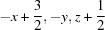
; (iii) 
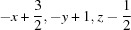
.

**Table 2:** Experimental details

Crystal data	
Chemical formula	C_17_H_18_N_2_O
*M*_r_	266.33
Crystal system, space group	Orthorhombic, *P* *b* *c* *a*
Temperature (K)	173
*a*, *b*, *c* (Å)	16.4313 (7), 7.1909 (3), 24.7345 (11)
*V* (Å^3^)	2922.5 (2)
*Z*	8
Radiation type	Mo *K*α
μ (mm^−1^)	0.08
Crystal size (mm)	0.24 × 0.22 × 0.18

Data collection	
Diffractometer	STOE IPDS II two-circle
Absorption correction	Multi-scan (*X-AREA*; Stoe & Cie, 2001 ▸)
*T*_min_, *T*_max_	0.742, 1.000
No. of measured, independent and observed [*I* > 2σ(*I*)] reflections	25768, 2574, 2200
*R*_int_	0.054
(sin θ/λ)_max_ (Å^−1^)	0.595

Refinement	
*R*[*F* ^2^ > 2σ(*F* ^2^)], *wR*(*F* ^2^), *S*	0.046, 0.111, 1.10
No. of reflections	2574
No. of parameters	276
No. of restraints	84
H-atom treatment	H atoms treated by a mixture of independent and constrained refinement
Δρ_max_, Δρ_min_ (e Å^−3^)	0.24, −0.21

Computer programs: *X-AREA* (Stoe & Cie, 2001 ▸), *SHELXS2014/7* and *XP* in *SHELXTL-Plus* (Sheldrick, 2008 ▸) and *SHELXL2014/7* (Sheldrick, 2015 ▸).

## supplementary crystallographic information

### Crystal data

**Table:**

C_17_H_18_N_2_O	*D*_x_ = 1.211 Mg m^−^^3^
*M**_r_* = 266.33	Mo *K*α radiation, λ = 0.71073 Å
Orthorhombic, *P**b**c**a*	Cell parameters from 24714 reflections
*a* = 16.4313 (7) Å	θ = 2.1–25.5°
*b* = 7.1909 (3) Å	µ = 0.08 mm^−^^1^
*c* = 24.7345 (11) Å	*T* = 173 K
*V* = 2922.5 (2) Å^3^	Block, colourless
*Z* = 8	0.24 × 0.22 × 0.18 mm
*F*(000) = 1136	

### Data collection

**Table:**

STOE IPDS II two-circle diffractometer	2200 reflections with *I* > 2σ(*I*)
Radiation source: Genix 3D IµS microfocus X-ray source	*R*_int_ = 0.054
ω scans	θ_max_ = 25.0°, θ_min_ = 2.1°
Absorption correction: multi-scan (X-Area; Stoe & Cie, 2001)	*h* = −18→19
*T*_min_ = 0.742, *T*_max_ = 1.000	*k* = −8→8
25768 measured reflections	*l* = −29→29
2574 independent reflections	

### Refinement

**Table:**

Refinement on *F*^2^	84 restraints
Least-squares matrix: full	Hydrogen site location: mixed
*R*[*F*^2^ > 2σ(*F*^2^)] = 0.046	H atoms treated by a mixture of independent and constrained refinement
*wR*(*F*^2^) = 0.111	*w* = 1/[σ^2^(*F*_o_^2^) + (0.0542*P*)^2^ + 0.639*P*] where *P* = (*F*_o_^2^ + 2*F*_c_^2^)/3
*S* = 1.10	(Δ/σ)_max_ = 0.001
2574 reflections	Δρ_max_ = 0.24 e Å^−^^3^
276 parameters	Δρ_min_ = −0.21 e Å^−^^3^

### Special details

**Table:**

Geometry. All esds (except the esd in the dihedral angle between two l.s. planes) are estimated using the full covariance matrix. The cell esds are taken into account individually in the estimation of esds in distances, angles and torsion angles; correlations between esds in cell parameters are only used when they are defined by crystal symmetry. An approximate (isotropic) treatment of cell esds is used for estimating esds involving l.s. planes.

### Fractional atomic coordinates and isotropic or equivalent isotropic displacement parameters (Å^2^)

**Table:**

	*x*	*y*	*z*	*U*_iso_*/*U*_eq_	Occ. (<1)
O1	0.63353 (8)	0.70433 (16)	0.38320 (5)	0.0509 (4)	
H1	0.6763 (14)	0.763 (3)	0.3660 (9)	0.075 (7)*	
N1	0.74117 (8)	0.41062 (18)	0.33137 (5)	0.0392 (3)	
C1	0.73647 (10)	0.4889 (2)	0.28545 (6)	0.0364 (4)	
H1A	0.6840	0.5185	0.2718	0.044*	
C2	0.66704 (11)	0.3836 (2)	0.36223 (7)	0.0470 (4)	
H2A	0.6668	0.2584	0.3789	0.056*	0.851 (4)
H2B	0.6190	0.3942	0.3383	0.056*	0.851 (4)
H2C	0.6278	0.3353	0.3353	0.056*	0.149 (4)
H2D	0.6797	0.2768	0.3861	0.056*	0.149 (4)
C3	0.66388 (15)	0.5357 (3)	0.40687 (9)	0.0350 (5)	0.851 (4)
H3	0.7194	0.5566	0.4222	0.042*	0.851 (4)
C4	0.60471 (12)	0.4859 (3)	0.45148 (8)	0.0394 (6)	0.851 (4)
H4A	0.6007	0.5912	0.4771	0.047*	0.851 (4)
H4B	0.5501	0.4654	0.4356	0.047*	0.851 (4)
N2	0.62980 (16)	0.3185 (3)	0.48085 (10)	0.0391 (5)	0.851 (4)
C5	0.57527 (15)	0.2327 (3)	0.50643 (8)	0.0361 (5)	0.851 (4)
H5	0.5210	0.2773	0.5038	0.043*	0.851 (4)
C21	0.5901 (2)	0.0675 (5)	0.54025 (17)	0.0352 (7)	0.851 (4)
C22	0.6613 (3)	−0.0353 (9)	0.5350 (3)	0.0425 (15)	0.851 (4)
H22	0.7005	−0.0016	0.5085	0.051*	0.851 (4)
C23	0.6751 (4)	−0.1864 (10)	0.5681 (3)	0.0544 (14)	0.851 (4)
H23	0.7232	−0.2582	0.5639	0.065*	0.851 (4)
C24	0.6186 (3)	−0.2338 (6)	0.6078 (2)	0.0561 (10)	0.851 (4)
H24	0.6295	−0.3338	0.6318	0.067*	0.851 (4)
C25	0.5473 (3)	−0.1359 (6)	0.61203 (15)	0.0503 (10)	0.851 (4)
H25	0.5080	−0.1711	0.6383	0.060*	0.851 (4)
C26	0.53203 (18)	0.0143 (5)	0.57820 (13)	0.0409 (7)	0.851 (4)
H26	0.4822	0.0805	0.5809	0.049*	0.851 (4)
C11	0.80792 (10)	0.5363 (2)	0.25222 (6)	0.0356 (4)	
C12	0.88687 (10)	0.4938 (2)	0.26791 (7)	0.0440 (4)	
H12	0.8961	0.4264	0.3004	0.053*	
C13	0.95204 (11)	0.5492 (3)	0.23646 (7)	0.0499 (5)	
H13	1.0058	0.5180	0.2472	0.060*	
C14	0.93971 (12)	0.6499 (2)	0.18938 (7)	0.0477 (4)	
H14	0.9848	0.6897	0.1683	0.057*	
C15	0.86123 (12)	0.6919 (2)	0.17337 (7)	0.0474 (4)	
H15	0.8522	0.7605	0.1411	0.057*	
C16	0.79573 (11)	0.6343 (2)	0.20430 (6)	0.0412 (4)	
H16	0.7419	0.6619	0.1927	0.049*	
C3'	0.6215 (9)	0.4977 (16)	0.3939 (5)	0.042 (3)	0.149 (4)
H3'	0.5637	0.4580	0.3990	0.050*	0.149 (4)
C4'	0.6731 (7)	0.4957 (14)	0.4447 (5)	0.040 (3)	0.149 (4)
H4'1	0.7295	0.5323	0.4356	0.049*	0.149 (4)
H4'2	0.6512	0.5870	0.4709	0.049*	0.149 (4)
N2'	0.6732 (9)	0.3067 (15)	0.4696 (4)	0.044 (3)	0.149 (4)
C5'	0.6203 (12)	0.283 (2)	0.5052 (6)	0.041 (3)	0.149 (4)
H5'	0.5840	0.3821	0.5129	0.049*	0.149 (4)
C21'	0.6110 (12)	0.104 (2)	0.5364 (9)	0.032 (4)	0.149 (4)
C22'	0.6731 (19)	−0.025 (5)	0.5415 (17)	0.035 (5)	0.149 (4)
H22'	0.7195	−0.0144	0.5188	0.042*	0.149 (4)
C23'	0.6693 (15)	−0.169 (5)	0.5788 (14)	0.039 (5)	0.149 (4)
H23'	0.7164	−0.2393	0.5873	0.047*	0.149 (4)
C24'	0.5946 (13)	−0.208 (4)	0.6038 (14)	0.048 (6)	0.149 (4)
H24'	0.5856	−0.3222	0.6219	0.057*	0.149 (4)
C25'	0.5353 (11)	−0.075 (3)	0.6010 (8)	0.034 (4)	0.149 (4)
H25'	0.4873	−0.0892	0.6221	0.040*	0.149 (4)
C26'	0.5437 (12)	0.079 (3)	0.5682 (9)	0.040 (4)	0.149 (4)
H26'	0.5019	0.1705	0.5676	0.048*	0.149 (4)

### Atomic displacement parameters (Å^2^)

**Table:**

	*U*^11^	*U*^22^	*U*^33^	*U*^12^	*U*^13^	*U*^23^
O1	0.0560 (8)	0.0352 (6)	0.0615 (8)	0.0078 (6)	0.0122 (6)	0.0145 (6)
N1	0.0464 (8)	0.0304 (6)	0.0409 (7)	−0.0005 (6)	0.0045 (6)	0.0015 (6)
C1	0.0401 (9)	0.0291 (7)	0.0402 (9)	0.0019 (6)	−0.0012 (7)	−0.0004 (6)
C2	0.0511 (10)	0.0366 (9)	0.0534 (10)	0.0021 (8)	0.0146 (8)	0.0083 (8)
C3	0.0352 (12)	0.0296 (10)	0.0401 (12)	−0.0021 (9)	−0.0030 (10)	0.0047 (9)
C4	0.0412 (13)	0.0369 (10)	0.0400 (10)	0.0021 (8)	0.0023 (8)	0.0030 (8)
N2	0.0373 (13)	0.0433 (11)	0.0367 (13)	−0.0005 (10)	0.0011 (11)	0.0064 (9)
C5	0.0352 (11)	0.0372 (11)	0.0360 (10)	−0.0012 (10)	−0.0007 (9)	−0.0028 (8)
C21	0.0364 (18)	0.0357 (14)	0.0334 (15)	−0.0080 (12)	−0.0046 (13)	−0.0033 (11)
C22	0.044 (2)	0.0458 (18)	0.038 (2)	−0.0041 (18)	−0.003 (2)	−0.0014 (17)
C23	0.066 (2)	0.044 (2)	0.053 (3)	0.0077 (14)	−0.0057 (16)	−0.003 (2)
C24	0.068 (3)	0.042 (2)	0.0583 (17)	−0.0111 (18)	−0.018 (2)	0.0112 (16)
C25	0.061 (2)	0.048 (2)	0.0417 (15)	−0.021 (2)	−0.0047 (15)	0.0085 (15)
C26	0.0428 (13)	0.042 (2)	0.0376 (19)	−0.0106 (14)	−0.0008 (11)	−0.0017 (15)
C11	0.0431 (9)	0.0272 (7)	0.0366 (8)	−0.0011 (6)	0.0013 (7)	−0.0019 (6)
C12	0.0458 (10)	0.0428 (9)	0.0435 (9)	−0.0006 (7)	−0.0010 (8)	0.0081 (7)
C13	0.0408 (10)	0.0540 (11)	0.0549 (11)	−0.0038 (8)	0.0022 (8)	0.0029 (9)
C14	0.0540 (11)	0.0424 (9)	0.0468 (10)	−0.0112 (8)	0.0105 (8)	−0.0020 (8)
C15	0.0643 (12)	0.0406 (9)	0.0373 (9)	−0.0027 (8)	0.0040 (8)	0.0043 (7)
C16	0.0486 (10)	0.0374 (8)	0.0377 (8)	0.0034 (7)	−0.0001 (7)	0.0005 (7)
C3'	0.046 (7)	0.030 (6)	0.050 (6)	−0.017 (5)	0.000 (5)	0.001 (4)
C4'	0.045 (7)	0.041 (5)	0.036 (6)	−0.007 (4)	0.007 (5)	−0.003 (4)
N2'	0.048 (7)	0.047 (5)	0.038 (5)	−0.004 (5)	0.001 (5)	0.002 (4)
C5'	0.046 (7)	0.040 (6)	0.035 (6)	−0.006 (5)	−0.003 (6)	0.000 (5)
C21'	0.039 (8)	0.033 (6)	0.024 (6)	−0.007 (5)	−0.004 (5)	−0.005 (5)
C22'	0.029 (7)	0.033 (6)	0.043 (9)	−0.007 (5)	−0.022 (6)	−0.012 (5)
C23'	0.036 (7)	0.035 (8)	0.046 (11)	0.006 (5)	−0.014 (5)	−0.006 (8)
C24'	0.042 (8)	0.029 (7)	0.073 (13)	0.010 (7)	−0.002 (8)	0.011 (6)
C25'	0.030 (6)	0.029 (8)	0.042 (10)	0.006 (5)	−0.006 (5)	0.005 (5)
C26'	0.047 (7)	0.030 (8)	0.042 (7)	0.003 (5)	−0.001 (5)	0.005 (6)

### Geometric parameters (Å, º)

**Table:**

O1—C3	1.436 (2)	C26—H26	0.9500
O1—C3'	1.522 (11)	C11—C12	1.388 (2)
O1—H1	0.93 (3)	C11—C16	1.394 (2)
N1—C1	1.270 (2)	C12—C13	1.382 (2)
N1—C2	1.451 (2)	C12—H12	0.9500
C1—C11	1.473 (2)	C13—C14	1.386 (3)
C1—H1A	0.9500	C13—H13	0.9500
C2—C3'	1.359 (13)	C14—C15	1.382 (3)
C2—C3	1.555 (3)	C14—H14	0.9500
C2—H2A	0.9900	C15—C16	1.384 (2)
C2—H2B	0.9900	C15—H15	0.9500
C2—H2C	0.9900	C16—H16	0.9500
C2—H2D	0.9900	C3'—C4'	1.515 (14)
C3—C4	1.513 (3)	C3'—H3'	1.0000
C3—H3	1.0000	C4'—N2'	1.492 (13)
C4—N2	1.465 (3)	C4'—H4'1	0.9900
C4—H4A	0.9900	C4'—H4'2	0.9900
C4—H4B	0.9900	N2'—C5'	1.249 (15)
N2—C5	1.259 (3)	C5'—C21'	1.508 (15)
C5—C21	1.473 (3)	C5'—H5'	0.9500
C5—H5	0.9500	C21'—C26'	1.367 (15)
C21—C22	1.390 (4)	C21'—C22'	1.382 (16)
C21—C26	1.392 (4)	C22'—C23'	1.389 (17)
C22—C23	1.380 (4)	C22'—H22'	0.9500
C22—H22	0.9500	C23'—C24'	1.402 (17)
C23—C24	1.392 (5)	C23'—H23'	0.9500
C23—H23	0.9500	C24'—C25'	1.364 (16)
C24—C25	1.372 (5)	C24'—H24'	0.9500
C24—H24	0.9500	C25'—C26'	1.384 (15)
C25—C26	1.389 (4)	C25'—H25'	0.9500
C25—H25	0.9500	C26'—H26'	0.9500
			
C3—O1—H1	108.2 (15)	C12—C11—C16	118.91 (15)
C3'—O1—H1	128.8 (16)	C12—C11—C1	122.55 (14)
C1—N1—C2	118.61 (15)	C16—C11—C1	118.49 (15)
N1—C1—C11	123.57 (15)	C13—C12—C11	120.21 (16)
N1—C1—H1A	118.2	C13—C12—H12	119.9
C11—C1—H1A	118.2	C11—C12—H12	119.9
C3'—C2—N1	133.2 (6)	C12—C13—C14	120.68 (17)
N1—C2—C3	107.89 (15)	C12—C13—H13	119.7
N1—C2—H2A	110.1	C14—C13—H13	119.7
C3—C2—H2A	110.1	C15—C14—C13	119.42 (16)
N1—C2—H2B	110.1	C15—C14—H14	120.3
C3—C2—H2B	110.1	C13—C14—H14	120.3
H2A—C2—H2B	108.4	C14—C15—C16	120.11 (16)
C3'—C2—H2C	103.9	C14—C15—H15	119.9
N1—C2—H2C	103.9	C16—C15—H15	119.9
C3'—C2—H2D	103.9	C15—C16—C11	120.65 (16)
N1—C2—H2D	103.9	C15—C16—H16	119.7
H2C—C2—H2D	105.4	C11—C16—H16	119.7
O1—C3—C4	105.89 (16)	C2—C3'—C4'	99.4 (10)
O1—C3—C2	108.42 (17)	C2—C3'—O1	114.7 (8)
C4—C3—C2	111.88 (17)	C4'—C3'—O1	94.6 (9)
O1—C3—H3	110.2	C2—C3'—H3'	115.1
C4—C3—H3	110.2	C4'—C3'—H3'	115.1
C2—C3—H3	110.2	O1—C3'—H3'	115.1
N2—C4—C3	112.03 (18)	N2'—C4'—C3'	110.6 (9)
N2—C4—H4A	109.2	N2'—C4'—H4'1	109.5
C3—C4—H4A	109.2	C3'—C4'—H4'1	109.5
N2—C4—H4B	109.2	N2'—C4'—H4'2	109.5
C3—C4—H4B	109.2	C3'—C4'—H4'2	109.5
H4A—C4—H4B	107.9	H4'1—C4'—H4'2	108.1
C5—N2—C4	116.9 (2)	C5'—N2'—C4'	114.6 (12)
N2—C5—C21	124.3 (2)	N2'—C5'—C21'	123.3 (14)
N2—C5—H5	117.9	N2'—C5'—H5'	118.3
C21—C5—H5	117.9	C21'—C5'—H5'	118.3
C22—C21—C26	119.6 (3)	C26'—C21'—C22'	117.3 (15)
C22—C21—C5	121.0 (3)	C26'—C21'—C5'	119.1 (15)
C26—C21—C5	119.4 (3)	C22'—C21'—C5'	122.8 (16)
C23—C22—C21	120.1 (4)	C21'—C22'—C23'	122 (2)
C23—C22—H22	120.0	C21'—C22'—H22'	119.2
C21—C22—H22	120.0	C23'—C22'—H22'	119.2
C22—C23—C24	120.1 (4)	C22'—C23'—C24'	118.8 (19)
C22—C23—H23	119.9	C22'—C23'—H23'	120.6
C24—C23—H23	119.9	C24'—C23'—H23'	120.6
C25—C24—C23	119.9 (3)	C25'—C24'—C23'	117.6 (17)
C25—C24—H24	120.1	C25'—C24'—H24'	121.2
C23—C24—H24	120.1	C23'—C24'—H24'	121.2
C24—C25—C26	120.5 (3)	C24'—C25'—C26'	121.2 (16)
C24—C25—H25	119.8	C24'—C25'—H25'	119.4
C26—C25—H25	119.8	C26'—C25'—H25'	119.4
C25—C26—C21	119.7 (3)	C21'—C26'—C25'	121.4 (15)
C25—C26—H26	120.1	C21'—C26'—H26'	119.3
C21—C26—H26	120.1	C25'—C26'—H26'	119.3
			
C2—N1—C1—C11	175.36 (14)	C1—C11—C12—C13	−176.91 (15)
C1—N1—C2—C3'	−79.8 (8)	C11—C12—C13—C14	0.9 (3)
C1—N1—C2—C3	−102.03 (18)	C12—C13—C14—C15	−1.2 (3)
N1—C2—C3—O1	81.51 (19)	C13—C14—C15—C16	0.2 (3)
N1—C2—C3'—O1	21.2 (14)	C14—C15—C16—C11	1.1 (3)
N1—C2—C3—C4	−162.08 (16)	C12—C11—C16—C15	−1.3 (2)
O1—C3—C4—N2	−177.91 (17)	C1—C11—C16—C15	176.04 (14)
C2—C3—C4—N2	64.2 (2)	N1—C2—C3'—C4'	−78.4 (8)
C3—C4—N2—C5	−158.7 (2)	N1—C2—C3'—O1	21.2 (14)
C4—N2—C5—C21	−176.9 (3)	C2—C3'—C4'—N2'	−66.3 (11)
N2—C5—C21—C22	−17.6 (6)	O1—C3'—C4'—N2'	177.7 (9)
N2—C5—C21—C26	162.1 (3)	C3'—C4'—N2'—C5'	−93.3 (14)
C26—C21—C22—C23	−1.6 (10)	C4'—N2'—C5'—C21'	−178.5 (15)
C5—C21—C22—C23	178.1 (6)	N2'—C5'—C21'—C26'	−169.5 (18)
C21—C22—C23—C24	−1.5 (12)	N2'—C5'—C21'—C22'	21 (4)
C22—C23—C24—C25	3.3 (11)	C26'—C21'—C22'—C23'	−3 (6)
C23—C24—C25—C26	−2.1 (7)	C5'—C21'—C22'—C23'	167 (4)
C24—C25—C26—C21	−0.9 (5)	C21'—C22'—C23'—C24'	14 (7)
C22—C21—C26—C25	2.8 (6)	C22'—C23'—C24'—C25'	−17 (6)
C5—C21—C26—C25	−176.9 (3)	C23'—C24'—C25'—C26'	10 (5)
N1—C1—C11—C12	2.0 (2)	C22'—C21'—C26'—C25'	−5 (4)
N1—C1—C11—C16	−175.34 (15)	C5'—C21'—C26'—C25'	−174.7 (19)
C16—C11—C12—C13	0.4 (2)	C24'—C25'—C26'—C21'	1 (4)

### Hydrogen-bond geometry (Å, º)

*Cg*1,*Cg*2, and *Cg*3, are the centroids of the C11–C16, C21–C26 and C21'–C26' rings, respectively

**Table:**

*D*—H···*A*	*D*—H	H···*A*	*D*···*A*	*D*—H···*A*
O1—H1···N1^i^	0.93 (3)	1.92 (3)	2.8430 (19)	174 (2)
C24—H24···*Cg*1^ii^	0.95	2.88	3.802 (5)	164
C15—H15···*Cg*2^iii^	0.95	2.96	3.796 (3)	148
C15—H15···*Cg*3^iii^	0.95	2.79	3.640 (12)	150

Symmetry codes: (i) −*x*+3/2, *y*+1/2, *z*; (ii) −*x*+3/2, −*y*, *z*+1/2; (iii) −*x*+3/2, −*y*+1, *z*−1/2.
